# Supplementary material for: A Patient-Specific Fracture Risk Assessment Tool for Femoral Bone Metastases: Using the Bone Strength (BOS) Score in Clinical Practice
Source: Cancers (Basel). 2022 Nov 29;14(23):5904. doi: 10.3390/cancers14235904 (PMC9740241; doi:10.3390/cancers14235904)
Supplement: Supplementary file 1 [file cancers-14-05904-s001.zip › cancers-1940866-supplementary/File S1 Supplementary material BOS report.pdf]

Supplementary material: example BOS report

BOS score
v2.0

Radboudumc
university medical center

BOS score Report – FemurA
for fracture risk prediction in femoral bone metastases

Patient FemurA has a BOS score of 4.8, which is lower than the threshold of 7.5, indicating a high fracture risk. Of the patients with a BOS score below 7.5, 33% fractured their femur within six months although the majority within two months (PPV), whereas 1% of the patients with a BOS score higher than 7.5 fractured their femur (NPV). Below, the BOS score of patient FemurA is depicted relative to patients in the BOS database.

Conclusion:
Patient FemurA has a high fracture risk.

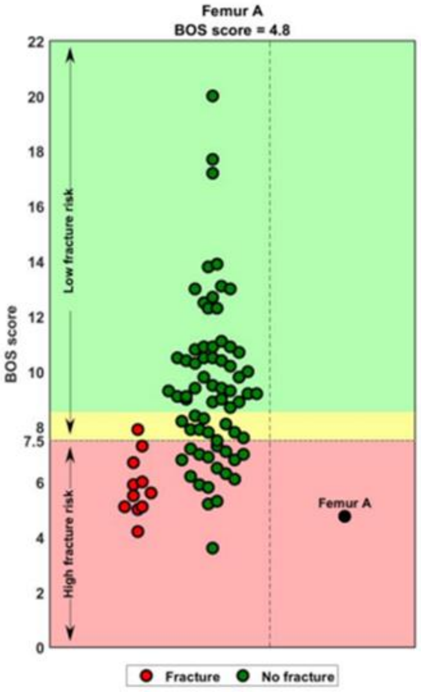

Figure for left-right check
The femur corresponding with the BOS score is indicated in green

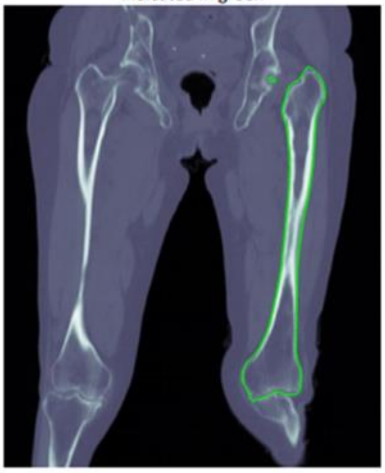

Weakest location of the bone
ATTENTION: Fracture localization by experimental fracturing in computer model, does not necessarily coincide with metastatic location

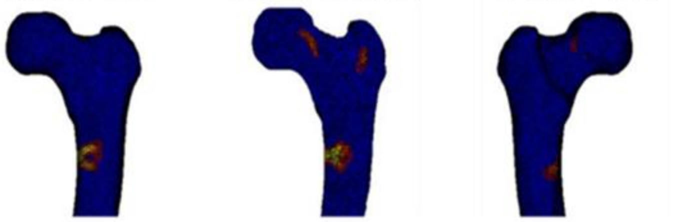

Value of the BOS score
Diagnostic values obtained from the patients in the BOS database: sensitivity, specificity, positive and negative predictive values are calculated using a threshold of 7.5 for distinguishing between high and low fracture risk:

Sensitivity = 93%	(% of fractured femurs that were correctly predicted as high-fracture risk)
Specificity = 73%	(% of non-fractured femurs that were correctly predicted as low-fracture risk)
Positive predictive value = 33%	(% of high-fracture risk femurs that indeed fractured)
Negative predictive value = 99%	(% of low-fracture risk femurs that did not fracture)

Data derived from Eggermont et al. 2018 Bone Joint Res; Eggermont et al. 2020 Bone, with additional results of the BOS study

Terms and conditions
No information provided in this report will give any guarantees. It is explicitly the responsibility of the physician to use and interpret the outcomes from this report correctly. Heath care providers should always also exercise their own independent clinical judgement when using the BOS score in conjunction with patient care.

Contact
orthopaedicresearchlab@radboudumc.nl
024 36 13366
