# Supplementary material for: A Patient-Specific Fracture Risk Assessment Tool for Femoral Bone Metastases: Using the Bone Strength (BOS) Score in Clinical Practice
Source: Cancers (Basel). 2022 Nov 29;14(23):5904. doi: 10.3390/cancers14235904 (PMC9740241; doi:10.3390/cancers14235904)
Supplement: Supplementary file 1 [file cancers-14-05904-s001.zip › cancers-1940866-supplementary/File S2 Supplementary material BOS database.pdf]

Supplementary material: BOS database (June 2021)

The current BOS database (n=114; 14 fractures) calculated with the most recent FE workflow. The four femurs that were prophylactically stabilized are also visualized, but are not included in the database since it is unknown whether they would have fractured without the surgery. Based on this database, the sensitivity of the BOS score is 93%, the specificity is 73%, the PPV is 33% and the NPV is 99%. Three patients (1 fracture, 2 non-fractures) with a knee prosthesis were excluded in this database due to failed femur segmentation when using the most recent FE workflow.

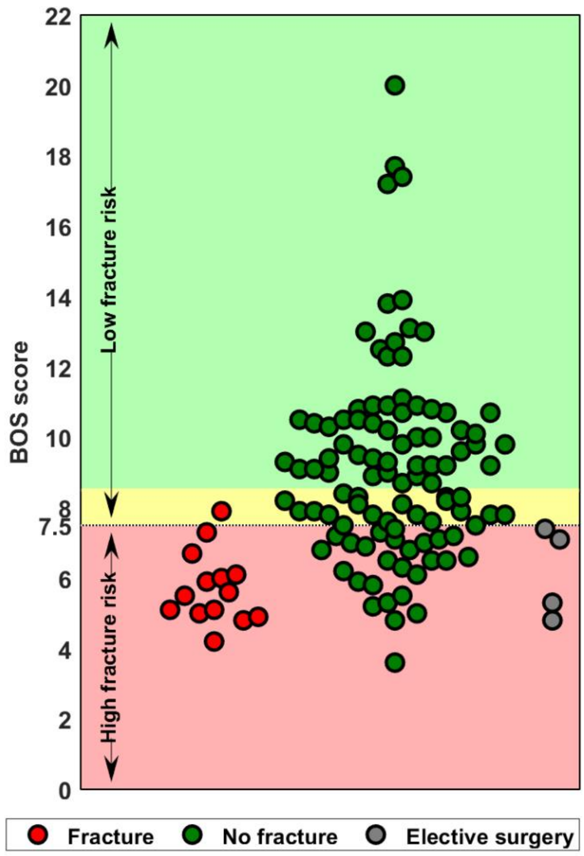

	Femurs that fractured during follow-up (n=14)	Femurs that did not fracture during follow-up (n=100)
BOS score ≤7.5 (high risk)	TP, n=13	FP, n=1
BOS score >7.5 (low risk)	FN, n=27	TN, n=73

Sensitivity = 93%	$\frac{TP}{TP+FN}$	Number of fractured femurs that were correctly predicted as high-fracture risk
Specificity = 73%	$\frac{TN}{TN+FP}$	Number of non-fractured femurs that were correctly predicted as low-fracture risk
Positive predictive value (PPV) = 33%	$\frac{TP}{TP+FP}$	Number of high-fracture risk femurs that did fracture
Negative predictive value (NPV) = 99%	$\frac{TN}{TN+FN}$	Number of low-fracture risk femurs that did not fracture

TP = true positives; TN = true negatives; FP = false positives; FN = false negatives
